# Matrix Metalloproteinases: Inflammatory Regulators of Cell Behaviors in Vascular Formation and Remodeling

**DOI:** 10.1155/2013/928315

**Published:** 2013-06-12

**Authors:** Qishan Chen, Min Jin, Feng Yang, Jianhua Zhu, Qingzhong Xiao, Li Zhang

**Affiliations:** ^1^Department of Cardiology, the First Affiliated Hospital, School of Medicine, Zhejiang University, 79 Qingchun Road, Hangzhou, Zhejiang 310003, China; ^2^Department of Reproductive Endocrinology, Women's Hospital, School of Medicine, Zhejiang University, 1 Xueshi Road, Hangzhou, Zhejiang 310006, China; ^3^Centre for Clinical Pharmacology, William Harvey Research Institute, Barts and the London School of Medicine and Dentistry, Queen Mary University of London, EC1M 6BQ London, UK

## Abstract

Abnormal angiogenesis and vascular remodeling contribute to pathogenesis of a number of disorders such as tumor, arthritis, atherosclerosis, restenosis, hypertension, and neurodegeneration. During angiogenesis and vascular remodeling, behaviors of stem/progenitor cells, endothelial cells (ECs), and vascular smooth muscle cells (VSMCs) and its interaction with extracellular matrix (ECM) play a critical role in the processes. Matrix metalloproteinases (MMPs), well-known inflammatory mediators are a family of zinc-dependent proteolytic enzymes that degrade various components of ECM and non-ECM molecules mediating tissue remodeling in both physiological and pathological processes. MMPs including MMP-1, MMP-2, MMP-3, MMP-7, MMP-8, MMP-9, MMP-12, and MT1-MMP, are stimulated and activated by various stimuli in vascular tissues. Once activated, MMPs degrade ECM proteins or other related signal molecules to promote recruitment of stem/progenitor cells and facilitate migration and invasion of ECs and VSMCs. Moreover, vascular cell proliferation and apoptosis can also be regulated by MMPs via proteolytically cleaving and modulating bioactive molecules and relevant signaling pathways. Regarding the importance of vascular cells in abnormal angiogenesis and vascular remodeling, regulation of vascular cell behaviors through modulating expression and activation of MMPs shows therapeutic potential.

## 1. Introduction

As blood vessels nourish all the tissues and organs in the body, it is unsurprising that abnormal formation and maintenance of blood vessels contribute to pathogenesis of numerous disorders. Indeed, excessive vascular formation causes cancer, arthritis, psoriasis and infectious disease, and so forth, whereas atherosclerosis, restenosis, hypertension, stroke, neurodegeneration, preeclampsia, respiratory distress, and osteoporosis are characterized by insufficient vessel growth or abnormal vascular remolding, and the list is still growing [1].

Vascular formation is coordinated in several modes comprising vasculogenesis—primitive vascular labyrinth assembly, angiogenesis—vascular sprouting and branching, and arteriogenesis—endothelial cell tubules covered by vascular mural cells [2–4]. After vessel maturation, enduring changes in the vascular composition and structure, known as vascular remodeling, occur in response to a number of stimuli. Vascular remodeling not only associates with vessel repair and adaptation, but also eventually affects luminal diameter and the thickness of vessel wall in every direction [5]. 

Both vascular formation and remodeling are complicated processes including recruitment, migration, proliferation, and apoptosis of vascular cells consisting of stem/progenitor cells, endothelial cells (ECs), vascular smooth muscle cells (VSMCs), and other mural cells. It is common that extracellular matrix (ECM) molecules play important roles in vessel development and morphogenesis by providing supportive matrix scaffold for cells, interacting with certain matrix receptors on cells and supplying growth factors that impact cellular function. Reciprocal interaction between vascular cells and their ECM is critical in blood vessel formation and remodeling.

Matrix metalloproteinases (MMPs) are a family of zinc-dependent proteolytic enzymes that degrade various components of ECM and mediate ECM remodeling in both physiological and pathological processes. A major function of MMPs is degradation of ECM to facilitate the progression of cell migration and invasion. However, a variety of research works reveal that proteolytic activity of MMPs controls availability of active molecules such as growth factors [6, 7]. Increasing evidence suggests that MMPs play a critical role in vascular formation and remodeling through degrading vascular basement membrane and ECM proteins and modifying angiogenic growth factors and cytokines. In normal physiological conditions, MMPs activities are regulated at multiple levels, including gene transcription, activation of zymogens and interaction with specific inhibitors in order to limit MMPs activity. Striking the balance enlarges MMPs activity and probably leads to pathological alterations in blood vessels [8].

The purpose of this review is to discuss the biological activities of MMPs in vascular formation and remodeling, especially to focus on increasing evidence elucidating the effects of MMPs on stem/progenitor cells, ECs, and VSMCs and their pathogenic role in related diseases.

## 2. MMPs: Regulators in Vascular Tissue

### 2.1. MMP Classification

MMPs are expanding family of endopeptidases first discovered in 1962 as a collagenase in resorption of the tadpole tail [9]. To date, the MMP family has at least 24 members, although some of them have not been well understood. Typical MMPs consist of a propeptide, a catalytic domain, a linker peptide (hinge region) and a hemopexin domain. The Zn^2+^ binding motif HEXXHXXGXXH in catalytic domain is the signature to assign proteinases to MMP family [6, 10]. 

MMPs are categorized by their structure and substrates into collagenases, gelatinases, stromelysins, matrilysins, membrane-type (MT)-MMPs, and others. Collagenases including MMP-1, MMP-8, MMP-13, and MMP-18 cleave interstitial collagen type I, II, and III and digest some of other ECM and non-ECM proteins. Gelatinases degrade both collagens and gelatins and include MMP-2 (gelatinase A) and MMP-9 (gelatinase B) which are the most widely studied MMPs in blood vessel. Stromelysins comprise MMP-3, MMP-10 and MMP-11. MMP-3 and MMP-10 have similar substrate specificities, and both digest wide range of ECM molecules and participate in proMMPs proteolysis, while the structure and function of MMP-11, also called stromelysin-3, diverge from other two stromelysins. Matrilysins which lack hemopexin domain include MMP-7 (matrilysin-1) and MMP-26 (matrilysin-2). MMP-7 processes many cell surface molecules, while MMP-26 degrades a number of ECM components. Membrane-type (MT)-MMPs consist of four transmembrane MMPs, MMP-14 (MT1-MMP), MMP-15 (MT2-MMP), MMP-16 (MT3-MMP), and MMP-24 (MT5-MMP) and two glycosyl-phosphatidylinositol-anchored MMPs, MMP-17 (MT4-MMP) and MMP-25 (MT6-MMP). MT-MMPs are expressed on the cell surface and activate proMMPs. Other MMPs that are not classified in previous categories include MMP-12, MMP-19, MMP-20, MMP-21, MMP-23, MMP-27, and MMP-28 [6, 10, 11].

Many studies of *in vitro* cultured cells and normal or diseased blood vessels reveal that both vascular cells and inflammatory cells in vessel wall can produce MMPs which participate in diverse vascular physiology and pathogenesis. To date, major MMPs expressed in vascular tissue and cell types include MMP-1, MMP-2, MMP-3, MMP-7, MMP-8, MMP-9, MMP-12, MMP-13, MT1-MMP, and MT3-MMP [8, 12].

### 2.2. MMP Activation and Function

Most MMPs are synthesized and secreted as inactive zymogens called proMMPs which have a cysteine switch motif PRCGXPD coordinating with Zn^2+^ in catalytic domain to maintain latency [13]. ProMMPs can be activated *in vitro* by chemical agents, such as thiol-modifying agents, oxidized glutathione, sodium dodecyl sulfate, chaotropic agents, and reactive oxygen species through the disturbance of cysteine-Zn^2+^ interaction of the cysteine switch [14]. However, stepwise activation of proMMPs *in vivo* is more complicated with removal of propeptide, disruption of cysteine-zinc binding, and detachment of the hemopexin domain, which is more likely conducted by other MMPs or other classes of proteinases such as plasmin and neutrophil elastase [7]. For example, proMMP-2 is activated by most MT-MMPs but not MT4-MMP [15]. 

Once activated, MMP catalytic domains contact with protein substrates and cleave at specific sites so as to breakdown the extracellular scaffold or modify biologically active molecules residing in ECM. In blood vessels, matrix scaffold degradation is mediated by activation of MMPs to facilitate endothelial and mural cell migration and invasion. In addition, various MMPs are necessary for releasing and processing of non-matrix molecules including growth factors such as fibroblast growth factors (FGFs), insulin-like growth factors (IGFs), transforming growth factor-*β* (TGF-*β*) and tumour necrosis factor-*α* (TNF-*α*) and their receptors, as well as integrins, plasminogen and adhesion molecules, and so forth, which impact vascular cell recruitment, proliferation, migration, and apoptosis in angiogenesis and vascular remodeling.

### 2.3. Vascular MMPs as Inflammatory Mediators

Vascular inflammation is a complex process encompassing multiple types of cells and various biological factors, which is initiated by tissue damage. Coordination of pro- and anti-inflammatory cytokines and chemokines regulates cell proliferation, adhesion, chemotaxis, migration, and apoptosis in inflammatory sites [16, 17]. In diseases related to angiogenesis and vascular remodeling, inflammation is identified and is always imbalanced, exacerbated, and chronic [18, 19].

There is complicated interplay between angiogenesis and inflammation. Angiogenesis sustains inflammation by providing metabolic demands to inflammatory cells, while inflammation promotes angiogenesis via releasing several cytokines and chemokines which impact vascular cell function and behavior [18]. Similarly, vascular inflammatory process is prominent in hypertension, atherosclerosis and restenosis, and so forth, in which abnormal vascular remodeling plays a predominant role [20]. During vascular remodeling, various cytokines are secreted from inflammatory cells such as monocytes, macrophages, and neutrophils, contributing to further recruitment of inflammatory cells and proliferation, migration, and apoptosis of ECs and VSMCs [17, 20].

MMPs function as such inflammatory cytokines during vascular formation or remodeling. In matured and quiescent vessels, active MMPs are absent or expressed at low levels. But in tissues undergoing abnormal angiogenesis and vascular remodeling, MMPs are markedly expressed, secreted, and activated [8]. Inflammatory cells such as macrophages and neutrophils are an important resource of MMPs in vascular tissue. Inflammatory factors, including tumor necrosis factor-alpha (TNF-*α*) and interleukins (ILs), stimulate MMP expression [21]. In turn, MMPs degrade ECM to facilitate migration and recruitment of cells including inflammatory cells and cleave cell surface receptors and other non-ECM molecules to mediate adhesion, proliferation, and apoptosis of cells in vessel wall which are involved in inflammatory process [22]. Therefore, MMPs are inflammatory mediators linking inflammation with angiogenesis and vascular remodeling.

### 2.4. Major Stimuli of Vascular MMPs and Their Contributions to Vascular Formation and Remodeling

**Figure 1:**
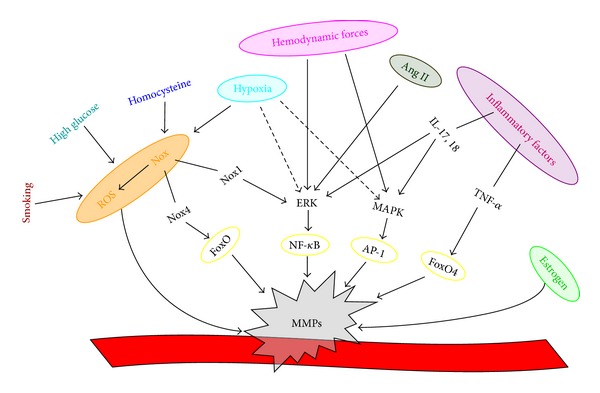
Major stimuli activating vascular MMPs.

Many studies investigating the relationship between MMPs and vascular biology demonstrate that a number of factors leading to angiogenesis and vascular remodeling-related diseases regulate MMP expression and activation, such as hemodynamics, oxidative stress, inflammation, hormonal factors, and hypoxia, (Figure 1).

Hemodynamic forces such as shear stress and arterial pressure regulate MMP expression and participate in vascular remodeling. Elevated transmural pressure activates MMP-2 and MMP-9 in *ex vivo* porcine carotid arteries [23]. In a murine model of blood flow cessation induced arterial remodeling, an early and significant increase in MMP-9 expression precedes the formation of intimal hyperplasia [24]. Low fluid shear stress induced MMP-9 expression involves integrins-p38 MAPK or ERK1/2-NF-kappaB signaling pathways [25]. Remodeling due to hemodynamic adaptation of the vein to the arterial condition leads to saphenous vein graft bypass failure. *Ex vivo* vein support system and *in vivo* animal model of vein graft reflect that high shear stress and increased venous pressure and wall tension induce overexpression of MMP-2 and MMP-9, which in turn drives vein graft wall remodeling [26–29].

Oxidative stress induced by imbalance between elimination and production of reactive oxygen species (ROS) has deleterious effect on vascular biology via excessive activation of MMPs. ROS formation via the NADPH oxidase (Nox) induced by mechanical stretch enhances MMP-2 mRNA expression and pro-MMP-2 release [30]. Oxidative stress induces the loss of retinal capillary cells by regulating the proapoptotic role of MMP-2 [31]. MMP-9 secretion and activity in monocytes are enhanced by increased Nox-dependent superoxide radical (^•^O_2_^−^) production in the atherosclerotic process [32]. In hindlimb ischemia, Nox2 derived ROS production increases MT1-MMP expression and MMP-9 activity, leading to neovascularization and tissue repair [33]. Moreover, ROS-dependent activation of MMPs is necessary for arteriolar inward remodeling [34]. Recent evidence also revealed the role of Nox in MMP transcription and activation. NOX4 induces MMP2 transcription via stimulating FoxO activity [35], while Nox1 in neointimal VSMCs initiates redox-dependent phosphorylation of ERK1/2 and subsequent MMP-9 activation following vascular injury [36]. In addition, a variety of other vascular MMP stimuli modify MMP functions via ROS-dependent ways. Homocysteine contributing to arterial remodeling induces increased activation of latent MMP-2 through an oxidative/nitrative dependent mechanism [37, 38]. High glucose stimulating MMP-1, MMP-2, and MMP-9 expression in cultured endothelial cells and macrophages is associated with ROS production [39–41]. Smoking-induced MMP activation promotes vascular remodeling through vascular inflammation and oxidative stress [42]. Regarding to the effects of oxidative stress in vascular remodeling, antioxidant approaches are used to reduce the upregulation of MMPs and attenuate the tissue remodeling during vascular diseases [43, 44].

Inflammatory process involving MMP activities is essential for the vascular remodeling, entailing reorganization of the ECM scaffold of the vascular wall, and particularly mediating atherosclerotic plaque progression and rupture [12, 21]. Inflammatory cells such as macrophages, mast cells, and neutrophils are the main sources of MMPs, and inflammatory cytokines like TNF-*α* and ILs stimulate MMP expression and activation [21, 22]. Taking TNF-*α*, IL-17 and IL-18 as examples, TNF-*α* activates MMP-9 gene expression at transcription level [45], while IL-17 and IL-18 also stimulate MMP-9 expression via nuclear factor-kappaB (NF-*κ*B) and activator protein 1 (AP-1) signaling activation [46, 47].

Hormonal stimuli including angiotensin II (Ang II), estrogen, and progesterone take part in vascular remodeling through activating vascular MMPs as well. Ang II stimulates MMP-8 and MMP-13 activity in atherosclerotic lesions, inducing intraplaque hemorrhages and plaque rupture via MMPs mediated degradation of interstitial collagen I [48]. Ang II also increases MMP-2 expression and activity in vascular remodeling during atherosclerosis, intimal and medial thickening, and hypertension [49–51]. In addition, an early increase in MT1-MMP expression with a subsequent increase in MMP-2 and MMP-9 activity has been observed in Ang II induced aneurysm formation [52], and MMP-9 production in aortic aneurysms relies on Ang II/ERK pathway [53]. Postmenopausal women receiving hormone replacement therapy are more likely to suffer from intimal hyperplasia after vascular intervention and bypass graft failure, which implies the significant role of estrogen and progesterone in vascular remodeling. Estrogen deficiency induced by ovariectomy gives rise to a reduction of active MMP-2 in the initial phase and a concurrent elevation of MMP-2 and MT1-MMP expression in latter period [54]. VSMCs incubated with estrogen and progesterone show upregulation of MMP pathway by increasing MMP-2 activity via enhancement of MT1-MMP expression [55], and inhibition of MT1-MMP prevents estrogen-stimulated increases in MMP-2 activity [56].

Hypoxia increasing expression levels of hypoxia inducible factor 1 (HIF-1) and vascular growth factors has a significant role in vasculogenesis, angiogenesis, and vascular remodeling [57, 58]. Hypoxia results in an overall upward tendency of vascular MMP-2 and MMP-9 expression. *In vitro* studies reveal that exposure of ECs to prolonged hypoxia enhances MMP-2 expression and activity [59, 60]. Consistently, rat exposed to hypoxia shows increased MMP-2 protein level and activity in aorta [61]. Chronic hypoxia also accelerates the development of atherosclerosis along with activated MMP-9 in apoE(−/−) mice [62]. The mechanisms of MMP upregulation by chronic hypoxia are not fully understood. However, hypoxia might regulate MMP expression transcriptionally due to its effect on activation of some transcriptional factors such as AP-1 and NF-*κ*B which are primarily involved in MMP gene expression [63, 64]. 

## 3. MMPs, Stem/Progenitor Cell Mobilization, Recruitment, and Angiogenesis

Multipotent adult stem/progenitor cells implicated in angiogenesis and vascular remodeling contribute to physiological and pathological processes through recruitment and migration into blood vessels and secretion of growth factors and cytokines. Bone marrow serves as a reservoir for several stem/progenitor cell populations, including mesenchymal stem cells (MSCs), endothelial progenitor cells (EPCs), and hematopoietic stem cells (HSCs) [65]. Another source of stem/progenitor cells involved in vascular diseases is blood vessel wall harboring MSCs, EPCs, and other stem/progenitor cells [66, 67]. Recruitment of stem/progenitor cells undergoes escaping from their niche, invading ECM and engrafting into target site where they proliferate and differentiate. To facilitate this mobilization, degradation of ECM via MMPs is an imperative requirement. MT1-MMP, MMP-2, MMP-9, and MMP-8 have been reported to function in stem/progenitor cell mobilization and recruitment in blood vessel formation and vascular remodeling (Table 1).

### 3.1. MT1-MMP in Bone Marrow-Derived Stem/Progenitor Cell Migration

Bone marrow-derived stem cells (BMSCs) induce an angiogenic effect and elicit vessel morphogenesis both *in vitro* and *in vivo* depending on proteolytic ability of MT1-MMP [68]. Lumican inhibits *in vitro* tube-like structure formation by bone marrow-derived MSCs via downregulation of MT1-MMP expression and activity in MSCs, and overexpression of MT1-MMP enhances MSC migration and invasion [69].

### 3.2. MMP-9 in Bone Marrow-Derived Stem/Progenitor Cell Mobilization

MMP-9 induced in bone marrow cells releases soluble Kit-ligand, allowing translocation of bone marrow EPCs and HSCs from quiescent bone marrow niche to proliferative vascular niche [70]. Deficiency of MMP-9 attenuates ischemia-induced neovascularization through an impairment of bone marrow-derived EPC adhesion, migration, and proangiogenic functions [71]. In tumor angiogenesis studies using MMP-9 knockout mice, transplanting tumors are unable to grow after irradiation in MMP-9 knockout mice, but tumor growth could be restored by transplantation of wild-type bone marrow. CD11b+ myelomonocytic cells rather than EPCs in transplanted bone marrow are responsible for immature vessels formation and tumor growth [72]. Moreover, it has been reported that elevated MMP-9 expression is associated with postinterventional restenosis [126]. Studies on coronary stent implantation patients reveal that active MMP-9 produced by neutrophils related to vascular injury possibly leads to mobilization of BMSCs (CD34+ stem cells), which may contribute to reendothelialization as well as restenosis [73, 74].

### 3.3. MMP-2 in Bone Marrow-Derived Stem/Progenitor Cell Mobilization

MMP-2, as another major vascular MMP, shows a significant role in normal and tumor angiogenesis and development of atherosclerotic and neointimal lesions [127–129]. These biological effects may attribute to a novel role of MMP-2 in stem/progenitor cells function. Bone marrow-derived EPCs (c-Kit+ stem cells) from MMP-2(−/−) mice display marked reduction in invasive and proliferative abilities and angiogenic responses. Ischemia-induced neovascularization in MMP-2(−/−) mice is impaired and can be restored by transplantation of bone marrow-derived mononuclear cells from MMP-2(+/+) mice [75], that suggests MMP-2 as a critical modulator of EPC mobilization and vascular formation.

### 3.4. MMP-8 in Vascular Stem/Progenitor Cell Migration

The pathogenic role of MMP-8 in progression and instability of atherosclerotic plaque has been discovered [130, 131]. We generated ApoE(−/−) MMP-8(−/−) double knockout mice and found substantially reduced extent of atherosclerotic plaque in these mice [131]. Furthermore, recent study from our group clearly shows that MMP-8 plays an important role in stem/progenitor cell migration and their recruitment from arterial lumen and adventitia into atherosclerotic plaque. ADAM10 cleavage and maturation by MMP-8 and subsequent E-cadherin shedding from cell surface could be the underlying molecular mechanism by which MMP-8 mediated stem/progenitor cell mobilization [76].

Previous studies elucidating the function of MMPs on stem/progenitor cells in blood vessels formation and vascular remodeling mainly emphasize on degradation of ECM promoting stem/progenitor cell mobilization and recruitment. However, MMPs are able to improve cell differentiation in other tissues [132–134]. Therefore, whether MMPs regulate proliferation and differentiation of stem/progenitor cells in vascular tissue via cutting and activating non-ECM molecules such as transcription factors needs further investigations.

## 4. MMPs and Vascular EC Function in Angiogenesis

Vascular ECs comprising a single layer of cells on the inferior surface of the blood vessels have unique and distinct function in vascular biology, especially in new blood vessel formation and angiogenesis. During vascular formation or angiogenesis, ECs are activated in response to environmental cues and express MMPs. Active MMPs degrade vascular basement membrane and other ECM resulting in detachment of ECs which is imperative in proliferation, migration, invasion, and even apoptosis of ECs and is an initial step of angiogenesis or vascular formation. In addition, MMPs can modify non-ECM molecules such as vascular endothelial growth factor receptors (VEGFR) and urokinase-type plasminogen activator receptor (uPAR) and their signaling pathways to affect EC vitality and behavior. Many MMPs have been shown to exert multiple impacts on ECs functions and angiogenesis (Table 2).

### 4.1. MMP-1/MMP-8 in Vascular EC Function and Angiogenesis

MMP-1 and MMP-8, two important collagenases, are highly active in vascular tissue and may sensitize EC function. Stimulation of ECs with active MMP-1 promotes expression of VEGFR2, a main binding receptor for VEGF-A and subsequent elevated endothelial proliferation through activation of protease activated receptor-1 (PAR-1) and NF-*κ*B pathway [77, 78]. Our most recent study provides strong evidence to suggest that MMP-8 plays an important role in EC angiogenesis *in vitro*, *ex vivo*, and *in vivo* by regulating the conversion of Ang I to Ang II, PECAM-1 expression, *β*-catenin nuclear accumulation, and cell proliferation and migration related gene expression [79].

### 4.2. MT1-MMP in Vascular EC Migration and Function

Evidence suggests that MT1-MMP affects EC migration and function. *In vitro* wound healing migration model reveals that expression of MT1-MMP is upregulated during EC migration and its activity can modulate endothelial migration, invasion, and formation of capillary tubes [80]. MT1-MMP contributes significantly to EC migration in both 2D collagen-coated surfaces and 3D collagen matrices, while secretory-type MMP-1, MMP-2, MMP-9, and MMP-13 are not critical for EC movement in 3D collagen gels [81]. In 3D collagen matrices, MT1-MMP-dependent collagen proteolysis is required in EC lumen formation and generation of vascular guidance tunnels that allow subsequent EC migration and tube network formation which is vital for blood vessel assembly [82]. MT1-MMP also acts as a key effector of nitric oxide (NO) in NO-induced EC migration and angiogenic processes via its collagenolytic function [83]. Furthermore, MT1-MMP performs not only as a matrix-degrading enzyme, but also a signaling molecule on ECs. MT1-MMP mediates small GTPases RhoA and Rac1 activation and subsequent Ca^2+^ and Nox-dependent signaling pathway, ultimately promoting tissue factor (TF) and plasminogen activator inhibitor-1 (PAI-1) protein expression, in thrombin-stimulated endothelial cells [135].

### 4.3. MMP-9/MMP-2 in Vascular EC Migration and Survival

MMP-9 and MMP-2, two gelatinases, have been shown as EC modulators. MMP-9 reduction is required for inhibition of invasion and angiogenesis in human microvascular ECs [84]. MMP-2 appears to be an essential molecule determining EC fate, paradoxical effects on both survival (angiogenesis) and cell death. Two major apoptotic pathways in ECs, caspases and p38 MAPK, enhance MMP-2 synthesis and affect EC behavior via different activation form of MMP-2. The partially active form supports survival and migration, while the fully active form leads to apoptosis, and eventually the ratio between these two MMP-2 activation forms in environment determines EC functions [59, 85].

### 4.4. MMP-12 in Vascular EC Proliferation and Migration

Studies in systemic sclerosis suggest that MMP-12 influences EC function and angiogenesis. Overexpression of MMP-12 cleaves urokinase-type plasminogen activator receptor (uPAR) and impairs uPA-dependent human microvascular EC proliferation, migration, and capillary morphogenesis in systemic sclerosis [86, 87]. Loss of function of MMP-12 in systemic sclerosis ECs restores the ability to induce vascularization [88].

### 4.5. MMP-7 in Vascular EC Proliferation and Migration

MMP-7, a matrilysin, accelerates the proliferation of human umbilical vein ECs *in vitro* and directly induces angiogenesis [89]. Moreover, MMP-7 impacts EC function through modulating VEGF pathway. Soluble VEGF receptor-1, an endogenous VEGF inhibitor via blocking VEGF access to membrane receptors, can be degraded by MMP-7, which liberates VEGF and enhances VEGF-induced VEGFR2 phosphorylation. The presence of MMP-7 finally promotes endothelial migration and tube formation via VEGF-VEGFR2 downstream activation in ECs [90].

## 5. MMPs and VSMC Behaviors

VSMCs are the major components of blood vessels. Abnormal VSMC proliferation, migration, and apoptosis are the main causes of vascular remodeling implicated in multiple vascular disorders, including hypertension, restenosis, and atherosclerotic plaque progression and rupture. Alterations of VSMC functions/behaviors also play important roles in physiological and pathological angiogenesis since VSMC and other mural cell recruitment is required for vessel formation and maturation. ECM degradation and remodeling indispensable to vascular structure alterations highlight MMP functions in VSMC behaviors. MMP-2, MMP-9, MT1-MMP, MMP-3, MMP-1, and MMP-7 have been recognized in vascular tissue and play pathogenic roles in vascular remodeling via regulating VSMC behaviors (Table 3).

### 5.1. MMP-2 in VSMC Proliferation and Migration

In cultured VSMCs, stimulation of MMP-2 production is related to mitogenesis and migration of VSMCs [91]. Pulmonary arterial hypertension (PAH) characterized by medial hypertrophy and ECM remodeling of pulmonary arteries is associated with MMP-2. Elevated MMP-2 is found in PAH arteries and its overexpression and activation stimulate VSMC proliferation leading to medial wall thickness [92, 93]. Oxidized LDL (ox-LDL), a risk factor promoting atherogenesis, induces VSMC proliferation through activating multiple pathways. MMP-2 triggers ox-LDL induced activation of sphingomyelin/ceramide pathway and subsequent ERK1/2 activation and DNA synthesis that finally leads to VSMC proliferation [94]. 

The effects of MMP-2 on VSMC migration have also been widely described. MMP-2 deficiency resulted in deceased arterial SMC migration and invasion ability *in vitro* and attenuated intimal hyperplasia after carotid ligation *in vivo* [95]. SMCs from saphenous vein (SV) have similar responses. Transfection SV-SMCs with MMP-2 siRNA resulting in MMP-2 silencing inhibits invasive capacity of cultured human SV-SMCs [96]. Young and aged human aortic SMCs possessing different migratory ability at least partially attribute to MMP-2 expression and activation. Young SMCs show higher migratory capacity due to producing more active MMP-2, while aged SMCs only produce inactive zymogen form of MMP-2 [97]. Moreover, many factors stimulate migration of VSMCs in MMP-2-dependent manner. For example, Interleukin-l*β* (IL-l*β*), an inflammatory cytokine which is related to VSMC migration during neointimal formation, enhances active MMP-2 synthesis and activation of pro-MMP-2, stimulating VSMC migration [98].

### 5.2. MMP-9 in VSMC Proliferation and Migration

Rat VSMCs overexpressing MMP-9 show enhanced migration and invasion in the collagen invasion assay as well as Boyden chamber *in vitro* and increased invasion into medial and intimal layers when seeded on the outside of the artery *in vivo* [101]. Genetic MMP-9 knockout impairs migratory activity of isolated VSMCs and decreases intimal hyperplasia [101, 102]. In addition, lack of MMP-9 reorganizes collagenous matrix and reduces VSMC attachment to gelatin [95, 102]. It indicates that MMP-9 not only degrades ECM, but also conducts a connection between cell surface and matrix.

Various cytokines induced in vascular injury and immunoinflammatory responses contribute to atherosclerosis and restenosis through MMP-9 mediated VSMC migration. TNF-*α* mediates VSMC migration and neointimal formation through upregulation of MMP-9. TNF-*α* upregulates nuclear FoxO4, which in turn activates transcription of MMP-9 gene, through the N-terminal, Sp1-interactive domain, and the C-terminal transactivation domain of FoxO4 [45]. Both IL-18 and IL-17 stimulate VSMC migration in an MMP-9-dependent manner. MMP-9 expression induced by IL-18 and IL-17 is via NF-*κ*B and AP-1 signaling activation [46, 47]. 

Studies also find that VSMC replication is significantly decreased in MMP-9(−/−) arteries [99]. However, there are limited data explaining how MMP-9 regulates cell cycle. Recent evidence shows that MMP-9 regulates VSMC proliferation by modulating cell adhesion as well as cadherin and *β*-catenin association. MMP-9 cleaves N-cadherin and releases *β*-catenin which translocates to the nucleus and regulates cyclin D1 expression in VSMCs [100].

### 5.3. MT1-MMP in VSMC Proliferation and Migration

MT1-MMP is initially described as an activator of MMP-2 in vascular remodeling. In neointimal development subjected to balloon catheter injury, increased MT1-MMP level is of importance to MMP-2 activation and neointimal formation [103, 104]. However, since MT1-MMP possesses a broad spectrum of substrates, an increasing number of studies demonstrate that MT1-MMP participates in vascular remodeling not only via MMP-2 activating.

MT1-MMP functions as a collagenase as well as a signaling molecule. Proteinases such as plasmin, cysteine proteinases, MMP-2, and MMP-9 previously linked to VSMC migration in 2D substrata do not play a vital role in 3D matrix environment, whereas MT1-MMP, a key pericellular collagenolysin of type I and III collagens, enhances VSMC invasion into 3D collagenous barriers *in vitro*. Furthermore, MMT1-MMP deficiency attenuates neointimal hyperplasia and arterial lumen narrowing *in vivo* via a MMP-2, and MMP-9-independent role [105]. On the other hand, MT1-MMP cleaves or modulates cell surface molecules to influence VSMC behavior. Platelet-derived growth factor (PDGF)/PDGFR*β* signaling pathway is critical in VSMC migration and proliferation during vasculature development and VSMC phenotypic switch [106, 107]. In VSMC investment of the vasculature, MT1-MMP associates with PDGFR*β* as a necessary cofactor. Active MT1-MMP functions as a PDGF-B selective regulator of efficient induction of PDGF-B/PDGFR*β* downstream signal transduction that eventually leads to proliferation and chemotaxis of VSMCs. MT1-MMP deficient mice display abnormal vessel wall morphology with notably reduced density of VSMCs [108]. Additionally, in VSMC dedifferentiation, MT1-MMP proteolytically processes LDL receptor-related protein 1 (LRP1) and promotes endocytosing of PDGFR*β*-*β*3-integrin-MT1-MMP-LRP1 complex, which impairs the negative regulation of PDGFR*β* depending on ligands binding to LRP1 [109, 110]. Moreover, ApoE is also shown to inhibit PDGF-induced VSMC migration and proliferation [111, 112]. This process is mediated via ApoE binding to LRP1 and subsequent activation of cAMP-dependent protein kinase A [113, 114]. Interestingly, apoE is identified as a MT1-MMP substrate, so that MT1-MMP can cleave apoE to suppress apoE-LRP1 binding and downstream signaling [115, 136]. To sum up, MT1-MMP-dependent LRP1 and apoE cleavage activate PDGF/PDGFR*β* signaling pathway and then enhance VSMC proliferation and migration.

### 5.4. MMP-1 in VSMC Proliferation and Migration

The effects of MMP-1 on VSMC migration and proliferation have been studied. Interstitial flow is elevated in vascular injury and hypertension and is believed to participate in VSMC migration and vascular remolding. In an *in vitro* 3D collagen I system, upregulation of MMP-1 mediates interstitial flow enhanced VSMC migration, while MMPs inhibitor GM6001 attenuates flow enhanced migration [116]. ERK1/2 phosphorylation and increased expression of AP-1 transcription factor c-Jun are implicated in interstitial flow induced MMP-1 expression and VSMC motility [117]. Additionally, studies of MMP-1 in PAH depict the preventive effect of MMP-1 in medial hypertrophy and enhanced remodeling of pulmonary arteries. MMP-1 transgenic mice show decreased medial hyperplasia via impaired cell proliferation of VSMCs and reduced excessive collagen deposition [118]. However, the mechanism underlying MMP-1 mediated impaired VSMC proliferation is still unclear.

### 5.5. MMP-3 in VSMC Proliferation and Migration

MMP-3, also known as stromelysin-1, influences saphenous vein SMC migration. The main cause of vein graft failure is intimal hyperplasia, a process dominated by proliferation and migration of VSMCs. MMP-3 overexpression significantly reduces SMC migration and inhibits neointimal formation in arterialized vein grafts [119]. Recently, a common insertion/deletion polymorphism in MMP-3 gene promoter region, known as 5A/6A polymorphism, is reported. There is evidence suggesting that 5A/6A polymorphism is related to MMP-3 expression and activation and individuals' susceptibility to many cardiovascular diseases [120]. However, MMP-3 5A/6A polymorphism does not affect invasion of saphenous vein SMCs isolated from patients with different genotypes [121].

It has been reported that MMP-3 regulates VSMC migration via MMP-9 activation. MMP-3 is already known as an activator of pro-MMP-9 [122]. MMP-3 knockout significantly reduces VSMC migration *in vitro* and neointima formation after carotid ligation *in vivo*. Combination of MMP-3 and MMP-9 knockout or knockdown reveals that MMP-3 mediated activation of MMP-9 is required and efficient in neointimal hyperplasia [123].

Preventive effects of MMP-3 in venous neointima via gene transfer and deteriorative role of MMP-3 found in carotid neointimal hyperplasia using genetic knockout seem paradoxical. The possible explanation could be the diverse properties of vein and artery, the distinct procedures inducing neointimal formation, or even the different methods generating genetic modifications. The fact that MMP-3 possesses the broadest substrate specificity among all MMPs makes it more complicated to fully understand the exact roles of MMP-3 in VSMC functions and vessel formation. 

### 5.6. MMP-7 in VSMC Apoptosis

Effects of MMPs on VSMC proliferation and migration are widely discussed in various studies. However, there areonly a few studies to investigate the functional involvements of MMPs in VSMC apoptosis. MMP-7, detectable in unstable atherosclerotic plaques, cleaves N-cadherin which is vital in cell adhesion and survival and then promotes VSMC apoptosis [124]. VSMC apoptosis leads to instability of plaque through thinning of fibrous cap [125]. Therefore, MMP-7-dependent N-cadherin cleavage and cell apoptosis may promote plaque development and rupture.

It has been shown that several cell survival and VSMC viability maintaining factors including N-cadherin, platelet-derived growth factor (PDGF), heparin binding endothelial growth factor (HB-EGF), and insulin-like growth factor 1 (IGF-1) can be cleaved and modulated by MMPs [137]. However, direct evidence presenting MMPs mediated VSMC apoptosis still needs to be achieved in the future work.

## 6. Conclusions and Perspectives

In conclusion, MMPs including MMP-1, MMP-2, MMP-3, MMP-7, MMP-8, MMP-9, MMP-12, and MT1-MMP play a crucial role in blood vessel formation, remodeling, or angiogenesis through regulating the functions or behaviors of stem/progenitor and vascular cells. Numerous stimuli which are risk factors of blood vessel-related disorders such as oxidative stress, inflammatory factors, hemodynamic forces, hormones, and hypoxia provoke MMP expression and activation. Once activated, MMPs modulate various behaviors of stem/progenitor cells, vascular ECs and VSMCs, which in turn contribute to physiopathological processes in vascular formation, remodeling as well as angiogenesis. The major and predominant role of MMPs in angiogenesis is still the degradation of ECM components to promote recruitment of stem/progenitor cells and facilitate migration and invasion of ECs and VSMCs. In addition, effects of MMPs on cell proliferation and apoptosis are discovered. It has been well known that other important molecular mechanisms by which MMPs regulate vascular cell proliferation and apoptosis are via proteolytically cleaving and modulating non-ECM molecules in VEGF-VEGFR signaling, uPA-uPAR signaling, and cell-cell adhesion.

As vascular cells are fundamental elements participating in vascular formation, remodeling, or angiogenesis, regulation of vascular cell behaviors via modulating the expression and activation of MMPs seems to provide a reasonable way for therapeutic purpose. A great number of MMP inhibitors have been tested experimentally. Tissue inhibitors of MMPs, inhibitory antibodies, and chemically-synthesized MMP inhibitors show certain effects on amelioration of pathological changes in animal models of vascular diseases [11, 138]. However, only doxycycline has been approved by the Food and Drug Administration for clinical application until now. This is partially due to the broad substrate spectrum, overlapped proteolytic effects, and wide distribution of MMPs and nonspecificity of MMP inhibitors. Several approaches such as site specific delivery and generating MMP inhibitors with increased selectivity are thought to be helpful for MMPs-targeted therapy. However, further understanding of MMPs governing vascular cell behaviors and their specific underlying mechanisms is still essential to develop novel therapies. Except for MMP inhibitors, factors promoting MMP gene transcription and signaling pathways mediating MMPs-induced vascular cell alterations could be potential therapeutic targets.

## Figures and Tables

**Table 1:** MMPs, stem/progenitor cell mobilization and recruitment, and angiogenesis.

MMPs	Stem/progenitor cells	Functions/effects	Substrates	Outcomes	References
MT1-MMP	BMSCs	Migration/invasion↑	ECM (fibrin)	Angiogenesis	[68, 69]

MMP-9	BM-EPCs/BM-HSCs (c-kit+)	Adhesion/migration↑	c-kit ligand	Neovascularization	[70, 71]
	BM-myelomonocytic cells (CD11b+)	Recruitment/invasion↑	ECM	Tumor vasculogenesis	[72]
	BMSCs (CD34+)	Migration/invasion↑	ECM	Restenosis	[73, 74]

MMP-2	BM-EPCs (c-kit+)	Invasion/proliferation↑	ECM VEGF signaling	Neovascularization	[75]

MMP-8	SPC (CD34+/c-kit+/Sca-1+/ Flk-1+)	Recruitment/migration↑	ECM ADAM10	Atherosclerosis	[76]

**Table 2:** MMPs and vascular EC function in angiogenesis.

MMPs	Functions/effects	Substrates/signaling pathway	Outcomes	References
MMP-1	Proliferation↑	PAR-1/NF-*κ*B→VEGFR2	Angiogenesis Vascular remodeling	[77, 78]
MMP-8	Proliferation/migration↑	Ang I→PECAM-1→*β*-catenin	Angiogenesis	[79]
MT1-MMP	Migration/invasion↑	ECM (collagen)	Vascular formation	[80–83]
MMP-9	Migration/invasion↑	ECM	Angiogenesis	[84]
MMP-2	Survival/apoptosis↑	Caspases and p38 MAPK	Angiogenesis Angioregression	[59, 85]
MMP-12	Proliferation/migration↑	uPAR	Systemic sclerosis	[86–88]
MMP-7	Proliferation/migration↑	Soluble VEGFR-1	Angiogenesis	[89, 90]

**Table 3:** MMPs and VSMC behaviors in vascular remodeling.

MMPs	Functions/effects	Substrates/signaling pathways	Outcomes	References
MMP-2	Proliferation↑	ET-1, sphingolipid signaling	Hypertension Atherosclerosis	[91–94]
	Migration/invasion↑	ECM	Atherosclerosis Neointimal hyperplasia	[95–98]

MMP-9	Proliferation↑	N-cadherin	Atherosclerosis Neointimal hyperplasia	[99, 100]
	Migration/invasion↑	ECM VSMC-ECM attachment	Atherosclerosis neointimal hyperplasia	[46, 47, 95, 101, 102]

MT1-MMP	Proliferation/migration↑	pro-MMP-2	Neointimal hyperplasia	[103, 104]
	Migration/invasion↑	ECM (collagen)	Neointimal hyperplasia	[105]
	Proliferation/migration↑	PDGF-PDGFR*β* Signaling (LRP1, ApoE)	Atherosclerosis Neointimal hyperplasia	[106–115]

MMP-1	Migration/invasion↑	ECM (collagen)	Neointimal hyperplasia	[116, 117]

MMP-1	Proliferation↓	Unclear	Preventive in pulmonary hypertension	[118]

MMP-3	Migration/invasion	ECM, pro-MMP-9	Neointimal hyperplasia	[119–123]

MMP-7	Apoptosis	N-cadherin	Atherosclerotic plaque instability	[124, 125]
